# NO Detection by Pulsed Polarization of Lambda Probes–Influence of the Reference Atmosphere

**DOI:** 10.3390/s131216051

**Published:** 2013-11-26

**Authors:** Sabine Fischer, Daniela Schönauer-Kamin, Roland Pohle, Maximilian Fleischer, Ralf Moos

**Affiliations:** 1 Department of Functional Materials, University of Bayreuth, Bayreuth 95440, Germany; E-Mails: Functional.Materials@Uni-Bayreuth.de (S.F.); Functional.Materials@Uni-Bayreuth.de (D.S.-K.); 2 Corporate Technology, Siemens AG, Munich 81739, Germany; E-Mails: Roland.Pohle@siemens.com (R.P.); Maximilian.Fleischer@siemens.com (M.F.)

**Keywords:** lambda probe, UEGO, NO sensor, NO_x_ sensor, mixed potential, YSZ, pulsed polarization, reference atmosphere

## Abstract

The pulsed polarization measurement technique using conventional thimble type lambda probes is suitable for low ppm NO_x_ detection in exhaust gas applications. To evaluate the underlying sensor mechanism, the unknown influence of the reference atmosphere on the NO sensing behavior is investigated in this study. Besides answering questions with respect to the underlying principle, this investigation can resolve the main question of whether a simplified sensor element without reference may be also suitable for NO sensing using the pulsed polarization measurement technique. With an adequate sensor setup, the reference atmosphere of the thimble type lambda probe is changed completely after a certain diffusion time. Thus, the sensor response regarding NO is compared with and without different gas atmospheres on both electrodes. It is shown that there is still a very good NO sensitivity even without reference air, although the NO response is reduced due to non-existing overlying mixed potential type voltage, which is otherwise caused by different atmospheres on both electrodes. Considering these results, we see an opportunity to simplify the standard NO_x_ sensor design by omitting the reference electrode.

## 1. Introduction

NO_x_ detection in the low ppm range for vehicle control or turbine emissions is of high interest since NO_x_ is a precursor for smog and acid rain. Due to that fact, various materials and sensing principles are under research, e.g., see reviews [1,2]. In addition to conventional sensors (potentiometric, impedancemetric, amperometric), novel concepts are under study like solid-state gas dosimeters that determine the accumulated amount of total NO_x_ in exhausts [3]. Besides conventional NO_x_ mixed potential type sensors based on yttria-stabilized zirconia [4–10], another new method uses conventional thimble type lambda probes (as described for instance in [11]) for NO_x_ sensing. Instead of operating continuously, a pulsed polarization technique is applied [12–14]. To further elucidate the sensing mechanism, the influence of the reference atmosphere on the sensor effect is of interest. If no air reference is required, a simpler sensor element without air reference but operated in the pulsed polarization technique might be feasible for NO_x_ detection. In order to clarify this, measurements are conducted using a thimble type lambda probe in a set up that allows one to change the reference, so that the same atmosphere is applied to both electrodes.

## 2. Experimental

### 2.1. Sensor Setup

**Figure 1.**
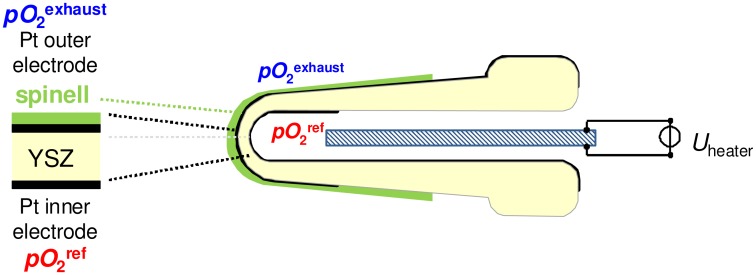
Cross section of a thimble type lambda probe.

Figure 1 shows the cross section of a conventional thimble type lambda probe. The two Pt electrodes on yttria-stabilized zirconia are exposed to different atmospheres, so that an oxygen sensor signal that follows the Nernst equation can be measured:

(1) $$U_{\text{Nernst}}=\frac{RT}{4e}\ln\frac{pO_{2}^{\text{exhaust}}}{pO_{2}^{\text{ref}}}$$

In Equation (1), *R* represents the universal gas constant, *F* is Faraday's constant, *T* stands for the absolute temperature, and *pO*_2_ is the oxygen partial pressure at the exhaust or reference side. At a minimum working temperature of 350 °C [11], oxygen ion conductivity and electrode kinetics are high enough for equilibrium oxygen sensing. The inner electrode is exposed to a reference atmosphere and the outer sensing electrode, which is coated with a protection layer made of spinel as shown in Figure 1, is exposed to exhaust gas. Thus the sensor has an asymmetric design.

**Figure 2.**
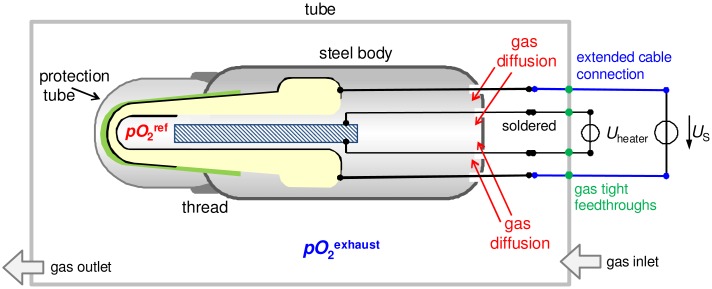
Setup to investigate the sensor response with changing reference atmosphere by gas diffusion.

To investigate the sensor response with and without reference atmosphere, the following setup was used (*cf*. Figure 2). The whole sensor with all cable connections was placed in a tube furnace. Because the cable connections are not high temperature stable, the sensor is heated only by the internal heater; and for that purpose the heating voltage *U*_heater_ was set to 14 V, whereas the furnace temperature remained at room temperature. Therefore, the tube of the furnace served only as a sample chamber. It is called “tube” in the following. The reference atmosphere of the thimble type lambda probe could be completely changed by gas diffusion through the non-gas-tight feedthroughs for cable connections in the lambda probe device, as depicted in Figure 2. According to this, it is expected that both electrodes can be exposed to the same gas atmosphere after a certain diffusion time.

### 2.2. Measurements

First, it was checked whether different (and equal) gas concentrations could be established on both sides. For that purpose, the diffusion time was evaluated by simply changing the oxygen content in the tube and recording the occurring voltage difference between inner and outer electrode with a sampling rate of 10 Hz. Nitrogen saturated with water served as a base gas (total gas flow 1 L/min). Oxygen was added alternately to the tube from 0.2 to 20 *vol*.% and from 2 to 20 *vol*.%, respectively. The results of this part are shown in Section 3.

**Figure 3.**
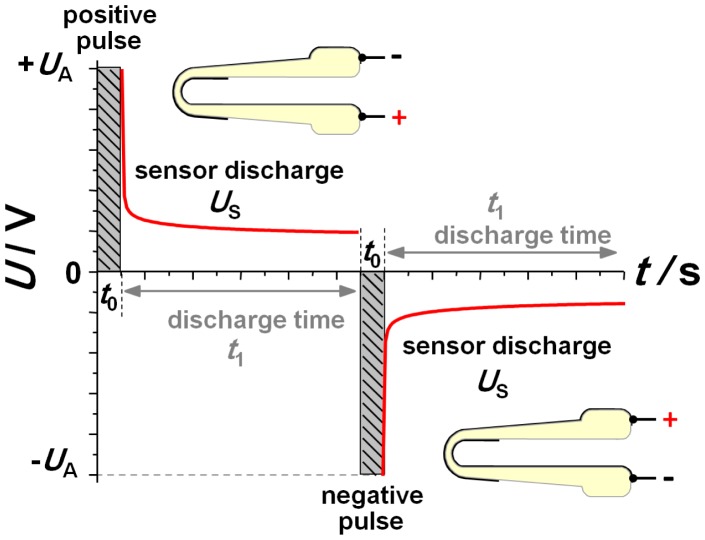
Scheme of the pulsed polarization technique (polarization parameter: *U*_A_ = 2.5 V, *t*_0_ = 1.0 s, *t*_1_ = 10 s) and the corresponding polarization sign of the electrodes.

After it was clear that equal as well as different gas concentrations could be established also on the inner side, the sensor response was investigated using pulsed polarization technique to obtain NO_x_ sensing behavior in the low ppm range [12–14]. Figure 3 illustrates this dynamic measurement technique consisting of alternating voltage pulses separated by discharge pauses. Amplitude *U*_A_ and polarization time *t*_0_ are the same for each voltage step. Only the sign is alternating. After each voltage pulse, the open circuit discharge voltage is measured during discharging (up to a discharging time *t*_1_). The meaning of the signs of polarization pulses as related to the design of the lambda probe are shown as insets in Figure 3.

During so called negative polarization voltage, the outer electrode of the sensor is on a higher potential so that the negatively charged oxygen ions move to the outer electrode. The polarity is verified by a comparison to Nernstian voltage resulting from an oxygen variation of the outer atmosphere. The optimum parameters of the polarization technique are a polarization amplitude of *U*_A_ = 2.5 V with a pulse duration of *t*_0_ = 1 s. Subsequently, the self-discharge voltage of the sensor was recorded for a duration of *t*_1_ = 10 s. The base gas during pulsed polarization measurements contained 10% oxygen, N_2_, and water as indicated above. NO was additionally dosed with a concentration of 50 ppm for 10 min.

## 3. Results and Discussion

### 3.1. Measurements to Evaluate the Diffusion Time into the Reference

In a regular operation mode, the thimble type lambda probe acts as an oxygen sensor. Its output follows the Nernst Equation (1). If the oxygen partial pressure, *p*O_2_^exhaust^, at the exhaust electrode is changed, a distinct voltage response, *U*_Nernst_, between both electrodes of the lambda probe forms, because the inner reference electrode potential is defined by the well-known reference atmosphere (usually air, with *p*O_2_^ref^ ≈ 0.2 bar).

**Figure 4.**
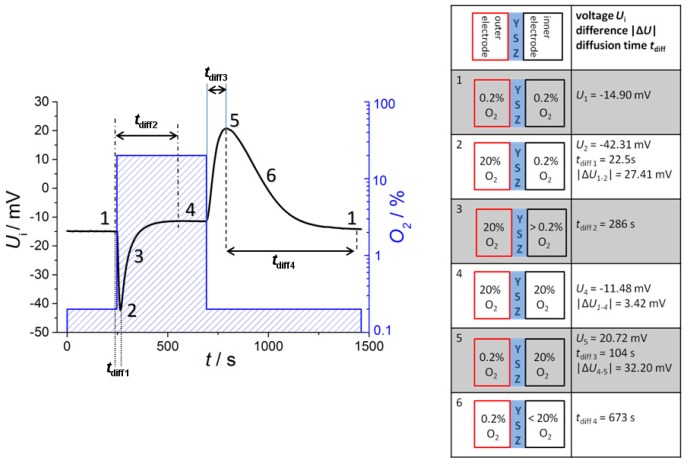
(**a**) Recorded voltage *U*_i_ and corresponding oxygen concentration. (**b**) Oxygen concentration at the outer electrodes during phase 1 to phase 6, and corresponding voltages *U*_i_, voltage differences |Δ*U_i-j_*|, and diffusion times *t*_diff i_ as indicated.

If one applies a new gas atmosphere, the voltage response can be explained by two different gas exchange processes: the exchange of the tube atmosphere governed by a gas flow of 1,000 mL/min (with a tube volume of about 0.3 liter) and the diffusion of the gas into the reference side, which is by far slower. Hence, one would expect first a rapidly changing sensor voltage due to the fact that the tube gets flushed with the new oxygen gas, *i.e.*, the exhaust side sees the new oxygen concentration, whereas the reference remains at the previous oxygen concentration. Then, oxygen diffuses slowly into (or from) the reference, so after some minutes, both electrodes face the same oxygen concentration. This expectation of the sensor behavior can be seen by the voltage readings and the corresponding oxygen concentrations in Figure 4. A discussion of the voltage response is given by dividing the signal into six phases with corresponding oxygen concentrations at the inner and outer electrode each.

At the beginning (phase 1), the oxygen concentration at both electrodes is the same (0.2%), and a constant voltage of *U*_1_= −14.90 mV is measured. This is astonishing at first sight, since according to Equation (1) one would have expected *U*_1_ = 0 V due to *p*O_2_^exhaust^ = *p*O_2_^ref^ ≈ 0.2 bar. However, oxygen ion conducting YSZ exhibits a Seebeck coefficient of around 500 μV/K [15], so that the measured offset voltage can be explained by a temperature difference of 30 K between both electrodes. This is due to the fact that the thimble type sensor is only heated by the internal heater, whereas the tube itself is at room temperature. At the heater voltage of *U*_heater_ = 14 V, the temperature of the inner electrode is almost 650 °C, so that 620 °C for the outer electrode seems to be realistic.

In phase 2, the oxygen concentration is changed stepwise in the tube by two decades from 0.2% to 20%. A maximum voltage of *U*_2_ = −42.31 mV is measured at *t*_diff1_ = 22.5 s. However, this voltage step |Δ*U*_1−2_| = 27.41 mV is markedly lower than the value calculated from Nernst equation. At 635 °C this value of |Δ*U*_1−2_| would yield an oxygen concentration ratio of 4.1. Supposing a rapid oxygen change including an oxygen concentration of quite 20% at the outer electrode, the maximum voltage response corresponds to a “real” oxygen content of about 5% at the inner electrode. This will be explained below. After *t*_diff1_ = 22.5 s the maximum voltage is recorded, which is in good accordance with the calculated diffusion time. The delay of *t*_diff1_ = 22.5 s can be understood easily if one takes into account that the gas exchange time of the tube is about 20 s (volume of about 0.33 liter; total gas flow 1 L/min).

After this peak, the voltage decreases due to gas diffusion through the feedthroughs of the leads to the inner electrode of the thimble type sensor (phase 3). The complete oxygen exchange is finished after *t*_diff2_ = 286 s and a constant voltage of *U*_4_= −11.48 mV occurs (phase 4). This base voltage deviates from the base line at 0.2% oxygen on both electrodes (phase 1). Caused by different temperatures of the two electrodes the Nernst Equation (1) has to be modified, because it is only valid for electrodes on the same temperature *T*. At various temperature levels *T*_outside_ and *T*_inside_ of the two electrodes following dependency of measured voltage from gas content is appropriate:

(2) $$U_{\text{measured}}=\frac{R}{4F}\ln\left(\ln pO_{2}^{\text{exhaust}}T_{\text{outside}}-\ln pO_{2}^{\text{ref}}T_{\text{inside}}\right)$$

with the same oxygen content *pO*_2_ = *pO*_2_^exhaust^ = *pO*_2_^ref^ at the electrodes. Assuming the same temperature difference Δ*T* = 30 K as before (phase 1) the base voltage at 20% oxygen is about 3 mV higher compared to 0.2% at both electrodes. The meaning of Equation (2) is equivalent with the *p*O_2_-dependency of the thermopower of YSZ of 49.6 μV/K per decade oxygen concentration [16]. At a temperature difference Δ*T* = 30 K the Nernst voltage needs to be corrected by further 2 × 49.6 μV/K × 30K = 2.98 mV. Thus the calculated voltage difference is nearly equal to the measured difference of |Δ*U*_1-4_| = 3.42 mV. It should be annotated here that Equation (2) can also be derived as a thermovoltage from irreversible thermodynamics [17].

In phase 5, the oxygen concentration is switched back to 0.2 % and a new voltage peak occurs after *t*_diff3_ =104 s with alternating sign (*U*_5_ = 20.72 mV). The voltage upon changing the oxygen at the outer electrode difference |Δ*U*_4−5_| = 32.20 mV is nearly 5 mV greater than at reverse oxygen step from low to high oxygen |Δ*U*_1−2_| = 27.41 mV. The difference between *t*_diff3_ and *t*_diff1_ can be explained if one considers that now the tube gas has to be exchanged with a low oxygen-containing gas.

Due to the decreasing oxygen gradient the voltage goes back in the following (phase 6) so that again a base voltage of nearly –15 mV occurs due to 0.2% oxygen on both sides after a long time of over 600 s (10 min). This baseline is equal to the voltage at the beginning of recording (phase 1).

**Figure 5.**
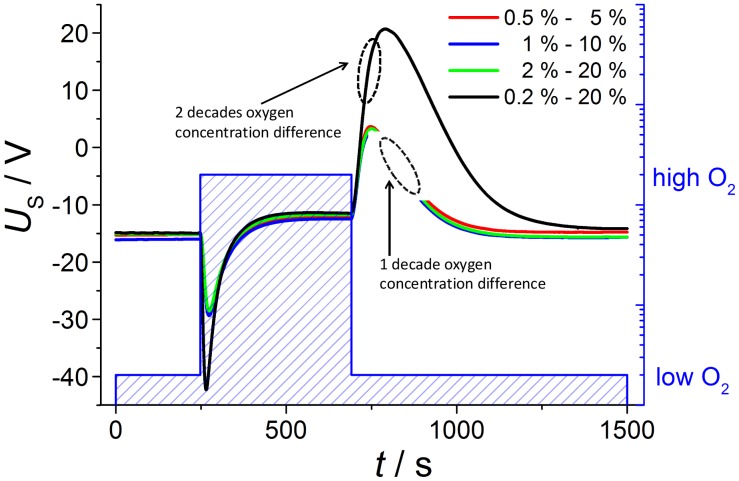
Voltage recording at changing oxygen concentrations with different concentration steps (one decade oxygen in contrast to two decades).

In order to further check the feasibility of whether it is possible to establish defined atmospheres also at the inner electrode, measurements are conducted with oxygen changes of one decade each (Figure 5); the low concentrations are 0.5%, 1% and 2% oxygen and the high contents are consequently 5%, 10% and 20%, respectively. According to Nernst Equation (1), the voltage step Δ*U* at oxygen switching should be halved compared to the previous measurement with an oxygen change of two decades from 0.2 to 20%. This prediction is verified by the measurements shown below.

The observed voltage dependencies are nearly the same for all investigated oxygen changes of one decade, so that both voltage amplitude and diffusion time are equal. The voltage step Δ*U* at oxygen switching from low to high oxygen is nearly 14 mV compared to 27.41 mV at measurement before with two decades of oxygen change. At the next gas change to low oxygen content the voltage shift is about 15 mV in contrast to 32.20 mV, so that the voltage peak is really halved according to the Nernst equation.

The observed voltage dependencies can also be estimated mathematically. If one assumes a temperature of 650 °C at the inner electrode and a temperature difference of 30 °C, one can estimate the thermovoltage, *U*_th_, that has to be added to the Nernst voltage acc. to Equation (1). If one further assumes a first order approach that the gas exchange in the tube from low to high oxygen concentrations, *c*_low_ and *c*_high_, respectively, can be expressed by an exponential Equation (3):

(3) $$c_{\text{exhaust}}\;(t)=\left(c_{\text{high}}-c_{\text{low}}\right)\cdot \left(1-\exp\left(-\frac{t}{\tau_{\text{tube}}}\right)\right)+c_{\text{low}}$$

and for the gas exchange in the tube from high to low oxygen concentrations:

(4) $$c_{\text{exhaust}}\;(t)=\left(c_{\text{high}}-c_{\text{low}}\right)\cdot \left(\exp\left(-\frac{t}{\tau_{\text{tube}}}\right)\right)+c_{\text{low}}$$

one can calculate the oxygen concentration at the exhaust electrode (tube facing electrode), *c*_exhaust_(*t*). In Equations (3) and (4), *τ*_tube_ is the tube time constant, roughly given by the volume of the tube (0.33 L) divided by the gas flow (1 L/min).

**Figure 6.**
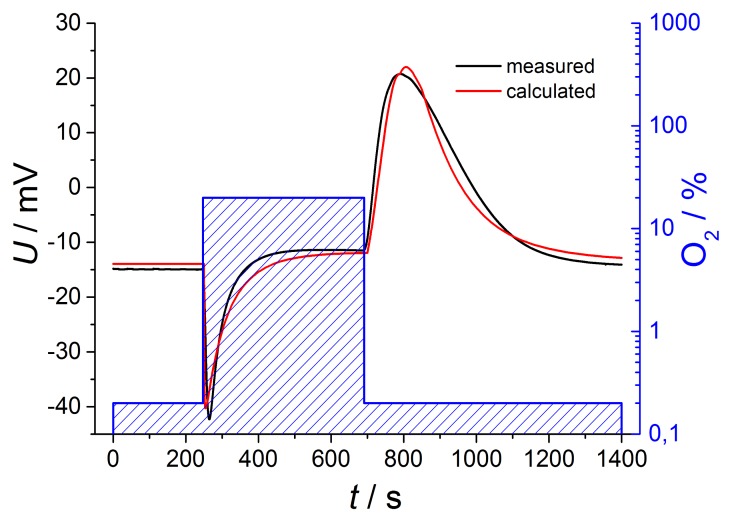
Calculated and measured voltages at two decades of oxygen change.

It is more difficult to estimate the concentration on the reference electrode, *c*_ref_(*t*). Here, we have to assume a diffusion process, with the difference of the concentrations in the tube and in the reference side as the driving force. However, since the concentration in the tube is not constant, one cannot find a closed expression for *c*_ref_(*t*). As a rough estimation, one may use also an exponential approach, but with another time constant, *τ*_reference_, that can be approximated experimentally. For an even better fit, one may use different time constants for the switch from high to low and from low to high oxygen concentrations. From the so-derived concentrations, *c*_exhaust_(*t*) and *c*_ref_(*t*), one can deduce the oxygen partial pressures and insert them into Equation (2) including different temperatures of both electrodes. If one adds the thermovoltage *U*_th_ to the Nernst equation *U*_Nernst_, the sensor voltage *U* can be calculated. Figure 6 compares the mathematical estimation with the measured voltages for oxygen changing of two decades.

The curves are in very good accordance regarding amplitude and shape, so it is possible to describe the sensor voltage in detail by this simple calculation. This exemplary description includes Nernstian equation considering temperature difference between both electrodes, oxygen dependent thermovoltage and various time constants for gas diffusion, but only in a first order approach.

### 3.2 Pulsed Polarization

After this preliminary study to verify that the atmosphere at the inner electrode can be changed by gas diffusion, the NO sensor response with and without reference atmosphere was evaluated by the pulsed polarization technique as described in Section 2.2 (polarization with 2.5 V for 1 s and discharging for 10 s). For this purpose, the discharge behavior at one gas concentration is exemplarily discussed in detail. After 600 s at base gas, NO is dosed with a concentration of 50 ppm for the same duration and a further base gas step follows to investigate whether the sensor response is changed due to prior NO dosing or not.

**Figure 7.**
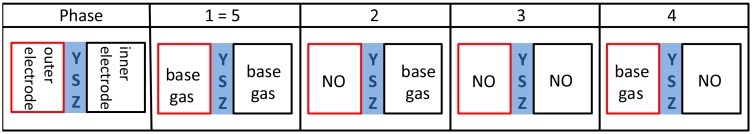
Different gas concentration on both electrodes during gas process including a NO step.

**Figure 8.**
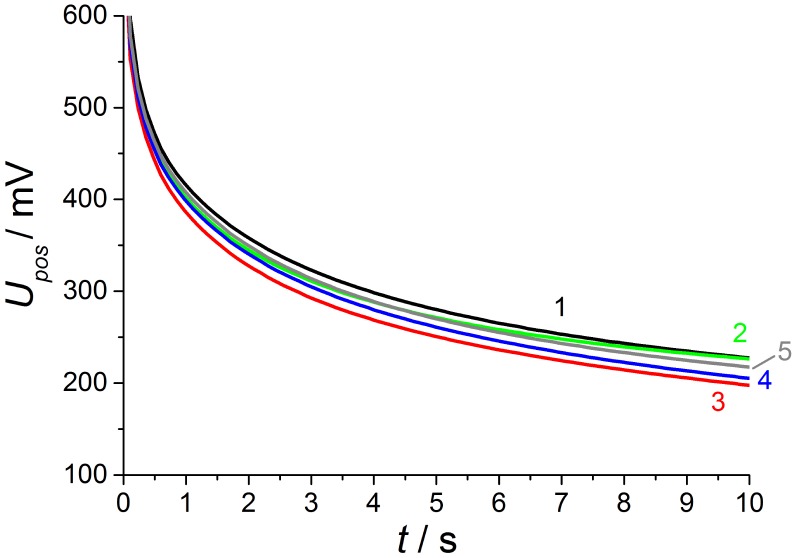
Discharge curves after ***positive*** (*cf.*Figure 3) voltage pulse at phases 1 to 5. The corresponding NO concentrations at the electrodes are shown in Figure 7.

**Figure 9.**
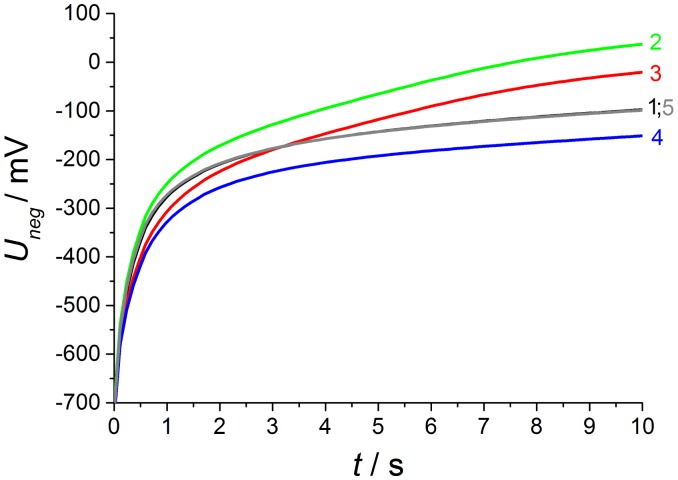
Discharge curves after ***negative*** (*cf.*Figure 3) voltage pulse at phases 1 to 5. The corresponding NO concentrations at the electrodes are shown in Figure 7.

By changing atmosphere and associated gas diffusion there are different phases of gas concentrations on both electrodes according to the preliminary study, which are shown in the gas process chart of Figure 7. The corresponding discharge curves are illustrated in the diagrams for both polarization signs at Figures 8 and 9.

All investigated discharge curves are nearly the same after positive voltage pulses, so that after polarization of 2.5 V for 1.0 s, the voltage drops to 800 mV directly after polarization and the voltage is still about 225 mV at the end of discharging. The highest voltage level after polarization, which is equivalent to the slowest discharge, is measured at base gas. Thus NO influences the self-discharge of the lambda probe only very slightly for positive signs. If NO is dosed to base gas, it is instantaneously present at the outer electrode and influences the discharge of the lambda probe, as can be seen by the slightly accelerated discharge of curve 2 compared to phase 1. This effect of only a few mV after positive voltage pulses is known from investigations before at conventional lambda probes [12,13].

With increasing time, NO diffuses into the thimble of the lambda probe so that the same NO concentration is present on both electrodes. The discharge curve at phase 3 is changed to more negative voltages, so that the discharge is accelerated compared to phase 1 and 2. The voltage difference to base gas on both electrodes (phase 1) amounts to 30 mV during the whole discharging period. This relatively high offset voltage cannot completely be explained by the temperature difference between inner and outer electrode and the associated different chemical potentials at the same NO containing atmosphere.

After turning off the NO dosing (phase 4), NO is only present at the inner (reference) electrode. This leads to a concentration difference with opposite sign compared to phase 2. The discharge voltage is at a higher level compared to phase 3, but the discharge is markedly faster as in phase 2 with opposite NO gradient. With increasing diffusion time, the discharge curve goes back to the base gas curve of phase 1 and only a small discrepancy is visible at the end of the diffusion time (phase 5).

As already shown in earlier publications, there is a markedly greater NO effect at opposite polarization pulses [12,13]. Both curve shapes and relaxation times differ from curves recorded after polarization with positive signs.

As shown in Figure 9, the discharge at base gas (phase 1) after negative polarization pulses starts at above *U*(0s) = −720 mV after a 2.5 V voltage pulse for 1 s. This is 80 mV less compared to the voltage directly after the opposite polarization pulse and the whole discharge curves are distinctly modified in shape and voltage level in contrast to the opposite polarization. Thus, the influence of different gas contents on both electrodes is markedly increased. At base gas, a voltage of about −120 mV is recorded at the end of discharge, which is almost the same voltage drop during the whole discharge process compared to the opposite polarization sign.

**Figure 10.**
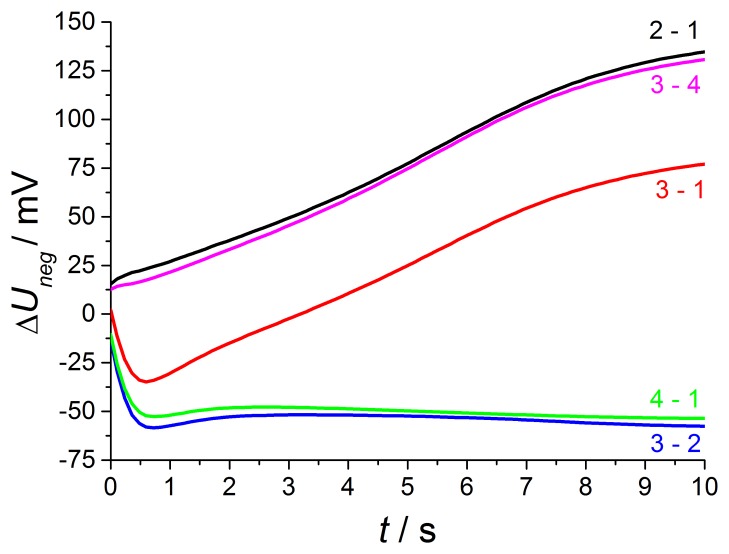
Voltage difference Δ*U* between different phases of NO presence on both electrodes.

As expected from [12,13], the NO effect on the discharge curve is by far larger compared to the discharge after positive voltage pulses. This is visible at curve 2 with NO only at the outer electrode and is equal to measurements using non-modified lambda probes with air reference electrodes. The discharge is markedly accelerated, so that the voltage after 10 s almost reaches *U*_10s_ = −40 mV in contrast to −120 mV at base gas on both electrodes. The presence of 50 ppm on the outer electrode causes a large voltage change, so that the voltage shift Δ*U*_2−1_ between phase 2 and phase 1 amounts to about 135 mV at the end of discharge time. The voltage differences Δ*U* between discharge curves recorded at different gas compositions on both electrodes are illustrated in detail in the following Figure 10.

With increasing diffusion time, the NO difference between both electrodes decreases in a way that at the end of the 600 s dosing period the same NO concentration appears on both electrodes (phase 3). The high voltage difference (Δ*U*_2−1_^max^ = 135 mV ) of phase 2 is reduced by presence of NO on both electrodes, but there is still a marked voltage change Δ*U*_3−1_^max^ of above 75 mV between phase 3 and 1 at the end of discharging, which is equal to a sensor without reference atmosphere. The curve shape of Δ*U*_3−1_ is equal to NO only at the outer electrode (Δ*U*_2−1_), but the amplitude of Δ*U*_3−1_ is constant about 60 mV lower compared to phase 2. This difference in discharging behavior can be explained by an additional constant offset voltage of 60 mV due to an existing NO gradient at phase 2 in contrast to phase 3 with the same NO containing atmosphere on both electrodes. As a conclusion, NO can be detected in spite of non-existing concentration gradients between the electrodes!

**Figure 11.**
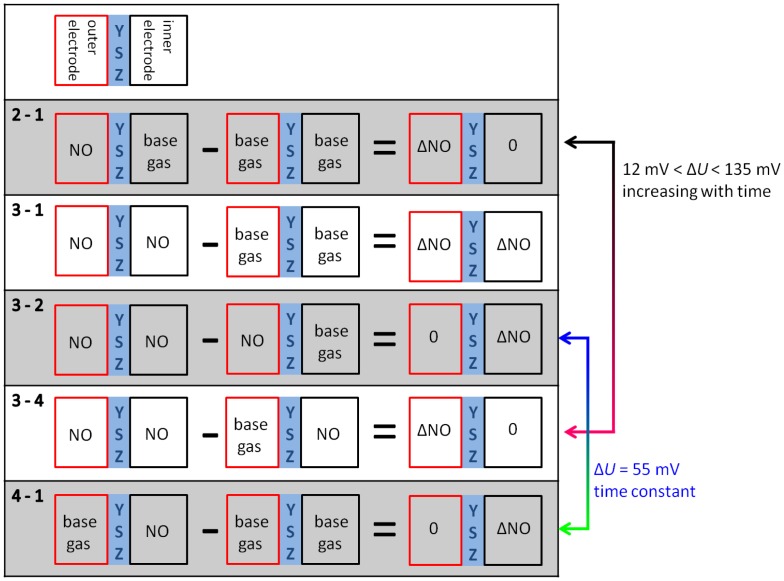
Illustration of the gas composition on both electrodes and the calculated differences according to Figure 10.

At phase 4—directly after the removal of NO—there is again a high NO gradient between the electrodes and NO occurs only at the inner electrode, whereas the outer electrode faces base gas only without any NO. This NO concentration gradient is opposite compared to phase 2, so that another discharge curve shown in phase 4 of Figure 9 is measured. The discharge curve is shifted to more negative values if NO is present at the inner electrode only. Therefore, the sensor discharges slower compared to base gas. The voltage difference Δ*U*_4−1_ between phase 4 and base gas (phase 5 and phase 1) is constantly 55 mV over the whole discharging time. This can be obtained from Figure 10. Hence, a constant offset voltage during the entire discharge process occurs. By comparing the voltage differences it is very prominent, that the Δ*U*-curves 2–1 and 3–4 are equal as well as 3–2 and 4–1, respectively. They correspond to the same calculated “gas differences” at the electrodes, which is shown explicitly in Figure 11.

Whether or not the gas difference with “zero value” stems from NO at the same electrode (Δ*U*_3−2_ with NO at the outer electrodes and Δ*U*_3−4_ at the inner electrode, respectively) or base gas at the same electrodes (Δ*U*_2−1_ with base gas at the inner electrodes and Δ*U*_4−1_ at the outer electrodes, respectively), there are the same difference curves, *i.e.*, Δ*U*_2−1_ = Δ*U*_3−4_ and Δ*U*_3−2_ = Δ*U*_4−1_.

A preliminary explanation is that different gas diffusion mechanisms occur at the inner and outer electrodes. Due to spinel coating of the outer electrode, there is time-dependent NO response. NO which is present at the outer electrode (Δ*U*_2−1_ = Δ*U*_3−4_) causes a timely increasing voltage difference Δ*U* from 12 mV at the beginning of the discharge up to a very high response of 135 mV at the end of the discharge period.

This is maybe caused by gas storage in this porous protection layer, which is also responsible for enhanced diffusion time at oxygen switching shown in the investigation before. Thus the large NO response is only measurable after long discharging times, when no additional oxygen is stored in this layer caused by negative polarization pulse and associated oxygen pumping to the outer electrode.

After the opposite polarization pulse, if oxygen ions are transported to the inner electrode without a similar layer, there is an almost timely constant NO effect. The offset voltage of about −55 mV occurring for 50 ppm at the inner electrode (Δ*U*_3−2_ = Δ*U*_4−1_) may originate from a mixed potential that forms immediately between the different electrodes. The electrodes differ in temperature as shown before and also in coating, so that mixed potential type response is measured and it does not depend on time, since no additional oxygen is stored at this electrode. All in all, there is a very different NO response after alternating polarization signs due to different sensor design, but the response is very high after both voltage pulses.

## 4. Conclusions and Outlook

By evaluating the voltage response to an oxygen changing atmosphere it is shown that the applied experimental setup is suitable for complete gas exchange of the inner reference atmosphere, so that after certain diffusion time the same gas concentration is present at both electrodes. Only an offset voltage is measured resulting from the temperature gradient between both electrodes. This leads to an additional thermoelectric voltage.

After this preliminary study, the pulsed polarization technique was applied to evaluate the NO sensitivity with and without reference atmosphere. It is well known from previous measurements at conventional thimble type lambda probes that this measurement technique is appropriate for NO_x_ sensing in the low ppm range, but it was unclear till now whether the air reference atmosphere is really necessary. Resulting from this evaluation, it is evident that NO sensing is possible even without reference electrode, but there are differences between the effects of NO on both electrodes due to the asymmetric sensor design. Thus, the NO effect at the outer electrode with spinel layer is enhanced compared to that at the inner electrode. Furthermore, the response depends on time, so that a long discharge time is necessary to obtain a high NO signal in contrast to evaluating the constant (but lower) NO response at the inner electrode.

As a consequence of these measurements, it seems feasible to design a planar sensor element without reference atmosphere by using pulsed polarization method for NO sensing in the low ppm range. Maybe a spinel layer amplifies the voltage response but longer discharge times may be necessary due to the timely increasing signal.

For some application in automotive exhaust gases the data acquisition time may be too long, *i.e.*, response times below a few seconds are required. However, from the results one gets the impression that the wide variety regarding the design of planar Pt ∣ YSZ sensors elements may enable the adaption of the discharging time constants in an optimum range, so that responses to NO_x_ changes below one second should be possible.

Additionally, a model to explain the special behavior of NO_x_ in contrast to all other exhaust gases is under development. Key issues are the thermodynamic gaseous equilibrium and the changes during electrode polarization as well as electrochemical reactions taking place at Pt ∣ YSZ systems under polarization. These considerations seem to be the basis of the high NO_x_ sensitivity and the very good sensor characteristics using pulsed polarization technique.
